# Cattle Uterus: A Novel Animal Laboratory Model for Advanced Hysteroscopic Surgery Training

**DOI:** 10.1155/2015/967693

**Published:** 2015-07-21

**Authors:** Ayman A. A. Ewies, Zahid R. Khan

**Affiliations:** Department of Obstetrics and Gynaecology, Birmingham City Hospital, Birmingham, West Midlands B18 7QH, UK

## Abstract

In recent years, due to reduced training opportunities, the major shift in surgical training is towards the use of simulation and animal laboratories. Despite the merits of Virtual Reality Simulators, they are far from representing the real challenges encountered in theatres. We introduce the “Cattle Uterus Model” in the hope that it will be adopted in training courses as a low cost and easy-to-set-up tool. It adds new dimensions to the advanced hysteroscopic surgery training experience by providing tactile sensation and simulating intraoperative difficulties. It complements conventional surgical training, aiming to maximise clinical exposure and minimise patients' harm.

## 1. Introduction

Advanced hysteroscopic surgical procedures such as transcervical resection of endometrium (TCRE), transcervical resection of polyp (TCRP), transcervical resection of fibroid (TCRF), and septum resection are minimally invasive procedures which have a slow learning curve and a narrow margin for error. Good training is conducive to sound practice, and the gynaecological surgeon will ultimately grasp the skills, hand-eye coordination, and manual dexterity to enable competent performance of these procedures in a shorter operating time [1].

Traditionally, surgical training in hysteroscopy takes place in the operating room where trainees first observe the procedure performed and then take on increasing roles in surgical cases under direct supervision [2]. However, the time required to perfect the required skills is no longer available to current generations of trainees given their overall shorter training period. With the implementation of the European Working Time Directive (EWTD), trainees in obstetrics and gynaecology now work a maximum of 48 hours per week, approximately half that of their predecessors, thus limiting their exposure to surgical procedures. Therefore, to minimise patient's harm and maximise surgical exposure, trainers are obliged to use other methods that replace real experience with ones that evoke or replicate substantial aspects of the real world in a fully interactive manner such as simulation or animal laboratories [3]. As early as 1927, Mayo, one of the founders of the renowned Mayo Clinic, argued that “there is no excuse for the surgeon to learn on the patients” [4]. The logical sequence of events is that one has to crawl before he or she can walk; therefore, skills need to be performed well and practiced thoroughly before one contemplates doing them on patients [1].

Apart from the “Pig Bladder Model” for training of basic hysteroscopic surgical skills and VersaPoint polypectomy [2], there is a paucity of soft tissue models that could be used for training of procedures such as TCRE, TCRP, TCRF, and septum resection. In this paper, we introduce the “Cattle Uterus Model” for advanced hysteroscopic surgery training in the hope that it will be adopted in various training courses in the UK and worldwide.

## 2. Materials and Methods

### 2.1. The Cattle Uterus

#### 2.1.1. Anatomy

**Figure 1:**
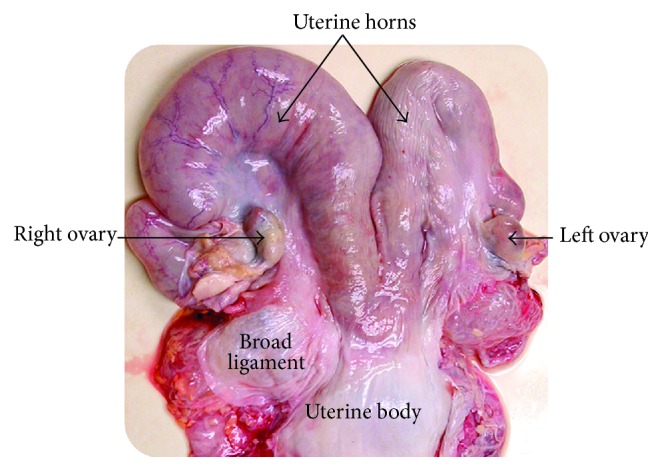
The anatomy of the cattle uterus.

The cattle uterus consists of a septate uterine body which is 4 to 5 cm long and two uterine horns 15 to 25 cm in length each (Figure 1). The uterus is suspended by the broad ligament in a coiled or curled manner. Each horn has its own oviduct. There is only one cervix which has 3–5 muscular cartilaginous transverse annular folds [5].

#### 2.1.2. Why Did We Choose the Cattle Uterus?

On exploring available options, various animals' uteri were considered inappropriate either because of the small size or having an anatomy which does not fit the purpose. The pig uterus, for example, consists mainly of two long horns 60–90 cm in length each with a small uterine body at the junction of both horns [6]. The advantages of using the cattle uterus as an animal laboratory model for the purpose of advanced hysteroscopic surgery training are the similarity in size of the uterine body with the human uterus, the realistic tissue resistance and tactile sensation that created a genuine training model which cannot be provided by the “Pig Bladder” model or simulation, and the presence of a uterine septum that allows training in septum resection, a surgical skill that is difficult to obtain given its rarity of occurrence in human.

#### 2.1.3. Supply

The cattle uteri are supplied by Wetlab-Medmeat (Kenilworth, Warwickshire, UK) which provides various types of tissue to aid surgical simulation. The products are derived from healthy animals which are intended for human consumption and humanely slaughtered in abattoirs in accordance with European Economic Community (EEC) regulations. These regulations under The Animal Health Veterinary Laboratories Agency (AHVLA) and EEC regulation 142/2011 allow for providing animal tissue material for educational purposes [7].

### 2.2. Developing the “Cattle Uterus Model”

The skills to perform advanced hysteroscopic surgery are often acquired by attending workshops that comprise didactic lectures and hands-on components with the aim of improving theoretical knowledge, enhancing clinical judgment, and initiating and upscaling manual dexterity [1]. We organised a three-day international hands-on advanced hysteroscopic surgery workshop at the Sandwell and West Birmingham Hospitals NHS Trust, UK. 14 consultants and senior trainees in gynaecology from the United Kingdom and overseas attended the workshop.

**Figure 2:**
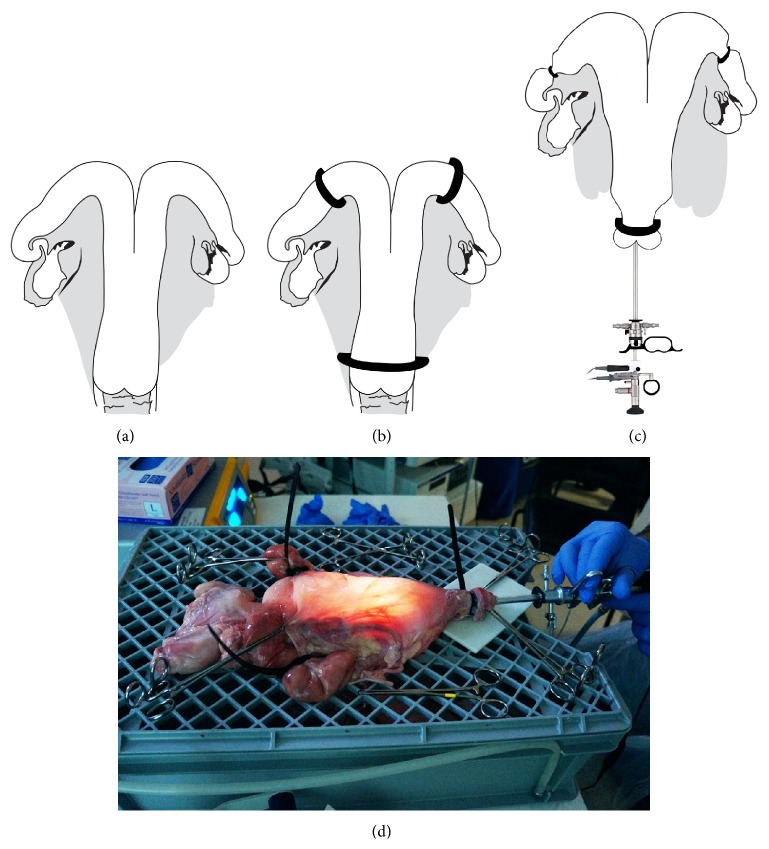
A diagram illustrating the shape of the cattle uterus and the site of placement of the 3 adjustable cable ties.

The “Cattle Uterus Model” was used for the first time where every candidate was trained on one uterus. The two uterine horns were clamped as close as possible to the uterine body using adjustable cable ties. The cervical canal of all uteri was wider than 10 mm; therefore, similar cable tie was used to squeeze the cervix to be fluid tight. Each uterus was fixed to a purpose built plastic crate using Allis forceps (Figures 2(a)–2(d)). The setup was then placed on top of a table with a pail below it to allow the saline to drain. The GYNECARE VERSAPOINT Bipolar Electrosurgery System (model number 00482, Cardiff, UK) and Olympus VISERA Elite Stack System (model number OTV-S190, Hamburg, Germany) were used to perform endometrial and septal resection.

## 3. Results

Candidates attending the course completed feedback questionnaires to provide an overall score (poor, average, good, or excellent) for each station and also to elaborate with free comments. Simulation of endometrial and septal resection using the “Cattle Uterus Model” turned out to be the most popular station and was rated excellent by 13 candidates and good by one. This was markedly better than Virtual Reality Simulator, which was rated as excellent and good by six and eight candidates, respectively. The free comments indicated that the participants favored the model that provided them with “a real feel of how it works.”

## 4. Comment

The “Cattle Uterus Model” offers a realistic platform to develop eye-hand-foot coordination and manual dexterity skills necessary for advanced hysteroscopic surgery. In addition, it offers simulated intraoperative difficulties for real-life events, for example, obscuration of vision due to floating endometrial chips or debris, overhang of the tissue to the wire loop, or saline bubbling. This allows trainees to practice useful manoeuvres to deal with them, for example, to flush out the debris by controlling the output channel and to remove bubbles by placing the scope into the bubble and opening the outflow channel. The only disadvantage of this model, and other animal laboratory models, is that they do not simulate the* in vivo* human condition as regards bleeding [1]. Therefore, we recommend it as a complement to the conventional surgical training and not as a substitute for apprenticeship or experience.

The traditional system of apprenticeship learning offers training under supervision in operating theatres allowing valuable one-to-one practical tuition in real-life situations. However, the implementation of EWTD for junior doctors decreased surgical exposure, which may present risks to the patients when the least experienced surgeon infrequently performs critical procedures using electrosurgery. Furthermore, there is evidence to suggest that teaching trainees in the operating room is challenging in terms of operating time and financial cost [1].

Visual Reality Simulators are used by the airline industry and military as well as in many medical specialties to educate, evaluate, and prepare for life-threatening scenarios [1]. They allow the opportunity for repeated practice, feedback, and ability to learn without causing harm. In addition, they are valuable in objectively scoring the trainee and assessing the learning curve with good reliability, validity, and cost-effectiveness [1, 2]. Nonetheless, in hysteroscopic surgery training, it is far from being realistic in terms of the inability to replicate actual tissue elasticity, resistance, and tactile sensation [2].

The Royal College of Obstetricians and Gynaecologists (RCOG) has implemented a structured “Advanced Training Skills Module” in hysteroscopic surgery, which has set several criteria for training and accreditation. It aims for senior trainees who would like to develop special interest in advanced hysteroscopic surgery. Hand-on workshops must be an integral part of professional development in advanced hysteroscopic surgery, and certainly the RCOG recommends that trainees attend such workshops as part of module completion [8]. We believe that using the “Cattle Uterus Model” will facilitate rapid acquiring of the necessary skills so that the trainees are able to finish the module within the proposed 12-month duration.

## Condensation

The “Cattle Uterus Model” offers a realistic platform for hands-on training in advanced hysteroscopic surgery, enabling efficient attainment of high level competency.
